# Retraction: Long non-coding RNA KCNQ1OT1 regulates cell proliferation, apoptosis and chemo-sensitivity through modulating the miR-186-5p/NCAM1 axis in acute myeloid leukemia cells

**DOI:** 10.1039/d1ra90012a

**Published:** 2021-01-22

**Authors:** Laura Fisher

**Affiliations:** Royal Society of Chemistry Thomas Graham House, Science Park, Milton Road Cambridge CB4 0WF UK advances-rsc@rsc.org

## Abstract

Retraction of ‘Long non-coding RNA KCNQ1OT1 regulates cell proliferation, apoptosis and chemo-sensitivity through modulating miR-186-5p/NCAM1 axis in acute myeloid leukemia cells’ by Jing Dai *et al.*, *RSC Adv.*, 2019, **9**, 36256–36265, DOI: 10.1039/C9RA06378A.

The Royal Society of Chemistry hereby wholly retracts this *RSC Advances* article due to concerns with the reliability of the data. The images in the article, and raw data provided by the authors, were screened by an image integrity expert. Analysis of the raw data showed that while the bands appeared to match the published figures, the backgrounds did not. The raw data, therefore, cannot be used to validate the published data. Furthermore, the raw data provided by the authors was found to closely resemble raw data for a number of other articles, which is unexpected given that there are completely different author lists for these articles.

In addition, the paper was analysed by experts who fact-checked the identities of the described nucleotide sequence reagents,^1^ and found errors with the following nucleotide sequence reagents reported in the article: miR-186-5p forward and reverse primers, and U6 reverse primer. Therefore, the results shown in Fig. 3 and 5 are unreliable.

In addition, the paper was analysed by another expert who found errors with the reagents and nucleotide sequencing reported in the article. Not all of the claimed identities of the sequences reported in the publication are correct for the reported RT-PCR primers, in particular the miR-186-5p forward and reverse primers, and U6 reverse primer, and therefore the results shown in Fig. 3 and 5 are unreliable.

Given the significance of the concerns about the validity of both the data in the article and the raw data provided by the authors, the findings presented in this paper are not reliable.

The authors have been informed but have not responded to any correspondence regarding the retraction.

Signed: Laura Fisher, Executive Editor, *RSC Advances*.

Date: 7^th^ January 2021.

## Supplementary Material
